# DINOSARC: Color Features Based on Selective Aggregation of Chromatic Image Components for Wireless Capsule Endoscopy

**DOI:** 10.1155/2018/2026962

**Published:** 2018-09-03

**Authors:** Michael D. Vasilakakis, Dimitris K. Iakovidis, Evaggelos Spyrou, Anastasios Koulaouzidis

**Affiliations:** ^1^Department of Computer Science and Biomedical Informatics, University of Thessaly, Lamia, Greece; ^2^Institute of Informatics and Telecommunications, National Center for Scientific Research “Demokritos”, Athens, Greece; ^3^Endoscopy Unit, The Royal Infirmary of Edinburgh, Edinburgh, UK

## Abstract

Wireless Capsule Endoscopy (WCE) is a noninvasive diagnostic technique enabling the inspection of the whole gastrointestinal (GI) tract by capturing and wirelessly transmitting thousands of color images. Proprietary software “stitches” the images into videos for examination by accredited readers. However, the videos produced are of large length and consequently the reading task becomes harder and more prone to human errors. Automating the WCE reading process could contribute in both the reduction of the examination time and the improvement of its diagnostic accuracy. In this paper, we present a novel feature extraction methodology for automated WCE image analysis. It aims at discriminating various kinds of abnormalities from the normal contents of WCE images, in a machine learning-based classification framework. The extraction of the proposed features involves an unsupervised color-based saliency detection scheme which, unlike current approaches, combines both point and region-level saliency information and the estimation of local and global image color descriptors. The salient point detection process involves estimation of DIstaNces On Selective Aggregation of chRomatic image Components (DINOSARC). The descriptors are extracted from superpixels by coevaluating both point and region-level information. The main conclusions of the experiments performed on a publicly available dataset of WCE images are (a) the proposed salient point detection scheme results in significantly less and more relevant salient points; (b) the proposed descriptors are more discriminative than relevant state-of-the-art descriptors, promising a wider adoption of the proposed approach for computer-aided diagnosis in WCE.

## 1. Introduction

Wireless Capsule Endoscopy (WCE) has now been established as a first-line diagnostic tool for small bowel diseases [1]. It makes use of a capsule endoscope (CE) which is a miniaturized, swallowable device, equipped with a miniaturized color video camera. Current CEs travel along the whole gastrointestinal tract (GI) by exploiting both gravity and its peristaltic movements. CEs are also equipped with a light source and transmit the captured images to an external recording device. Typically, a commercially available CE is able to capture approximately 100 K images during its journey allowing therefore collection of rich information about the GI tract in a minimally invasive manner. Since this amount of visual content is substantial, WCE video reading is a time-consuming process, prone to human errors, and it has been shown that detection rate of lesions is approximately 40% [2]. To overcome this, many research efforts have turned towards fully automatic analysis of WCE videos [3] and have been successfully applied on the problem of diagnosis of GI lesions, aiming to recognize several kinds of abnormalities, such as ulcers, polyps, and bleeding.

The majority of research works in WCE and in the broader field of endoscopic image analysis focuses on the interpretation of their visual content by either classifying them to normal/abnormal or further characterizing the abnormal ones according to the pathology of the depicted abnormality [4]. Image classification methodologies proposed in the context of automatic WCE image analysis include mainly supervised approaches addressing the detection of only a single or a few kinds of abnormalities [5], for example, polyps, ulcer, and bleeding, whereas fewer have addressed the detection of various kinds of abnormalities [6–8].

Many WCE image analysis approaches begin by detecting salient points to possible regions of abnormalities. Iakovidis and Koulaouzidis [6] proposed the use of the Speeded Up Robust Features (SURF) [9] algorithm on the chromatic component *a* of CIE-*Lab* to detect such salient points. From each salient point, color descriptors were extracted to characterize it along with its local neighborhood. These include the CIE-*Lab* values of the salient point and the minimum and maximum values of a square window centered at this point. This approach was very effective in the detection of a total of 9 different kinds of abnormalities. The success of this method was attributed in the physical meaning of these features which encodes more robustly the differences between normal and abnormal tissues.

Most of the current WCE image analysis methods aim to detect bleeding. In this context, the method proposed in [9] was applied for the detection of salient superpixels corresponding to bleeding regions. Following a Correlation-based Feature Selection (CFS), best discrimination of the superpixels containing blood was achieved using a three-dimensional vector of central moments estimated from *a* and saturation. In a later study [10], another bleeding detection method was based on saliency maps generated by fusing color component *a* of the CIE-*Lab* color space, component *M* of the *CMYK* color space, and the similarity to the red color values, that is, if a pixel has a greater *R* value and smaller *G* and *B* values, it would seem reddish and should be assigned higher saliency value. Other saliency detection methods exploiting saliency at a regional level for bleeding detection include the works of Fu et al. [11] and Shi et al. [12].

Fewer methods have been proposed for the detection of other kinds of abnormalities. Most challenging ones are those addressing polypoid lesion detection. Polypoid lesions are visible tissue masses protruding from the mucosal surface. They are characterized according to their color, appearance of their mucosal surface, presence of ulceration(s), their bleeding tendency, and the presence of pedunculus. In the context of polypoid lesions detection, most previous studies have been performed on images of flexible (conventional) endoscopy [13]. In a relevant study, Bernal et al. [14] proposed the Window Median Depth of Valleys Accumulation (WM-DOVA) energy maps, which were related with the likelihood of polyp presence and used them for polyp detection. Li and Meng [15] proposed a tumor detection system for WCE images, which exploited textural features based on Uniform Local Binary Pattern (ULBP) and wavelet transform. Recently, Yuan et al. [16] proposed a polyp detection approach for WCE. It was based on the Scale Invariant Feature Transform (SIFT) algorithm for the detection of salient points and the extraction of features using the SIFT descriptor and Complete Local Binary Pattern (CLBP). Several other relevant applications have been proposed for other kinds of abnormalities. For example, in [17] our earlier approach [6] was applied for weakly-supervised inflammatory lesion detection. The machine-learning approach used is called weakly-supervised because the images used for the training of the classifier need not be annotated in detail (pixel-by-pixel); instead, only keywords semantically describing their content are sufficient. In that study, weak supervision was achieved by the Bag of Visual Words (BoVW) model [18]. This model enables the representation of entire WCE images using histograms of visual words.

In this paper, we consider the saliency of a WCE image as the main component that can improve the discrimination of abnormal from normal WCE images, and we propose a novel feature extraction methodology that extends our previous approaches [6, 7, 19] for the detection of various kinds of abnormalities in WCE images. This methodology includes an unsupervised salient point and region detection algorithm and the estimation of local and global image descriptors enabling the characterization of various abnormalities both at a regional and at an image level. It consists of several novel components, including a color-based salient point detector, a salient region detector defining salient superpixels, and a method to derive a vectorial representation of the color of the salient superpixels by taking into account both point and region-level information. This enables more accurate localization and characterization of even very small abnormalities. Besides the local image descriptors derived, global image descriptors are derived for weakly-supervised abnormality detection based on a BoVW model.

The rest of this paper is organized in four sections. In Section 2, we describe the materials of this study, and in Section 3, we present the methodology proposed in this study. In Section 4, we evaluate the proposed methodology in comparison to relevant state-of-the-art approaches and discuss the obtained results. In the last section, we summarize the conclusions that can be derived from this study.

## 2. Materials

The research performed in this study was based on a rich and diverse collection of WCE images that include a variety of abnormalities and normal images from various parts of the GI tract. We provide this collection through an online database, called KID [20]. KID is composed of thousands of WCE images obtained from the whole GI tract using a MiroCam capsule endoscope with a resolution of 360 × 360 pixels. Abnormalities depicted within this dataset include 303 vascular (small bowel angiectasias and blood in the lumen), 44 polypoid (lymphoid nodular hyperplasia, lymphoma, Peutz–Jeghers polyps) and 227 inflammatory (ulcers, aphthae, mucosal breaks with surrounding erythema, cobblestone mucosa, luminal stenoses and/or fibrotic strictures, and mucosal/villous oedema) lesion images and 1,778 normal images obtained from the esophagus, the stomach, the small bowel, and the colon.

## 3. DINOSARC Feature Extraction

The proposed feature extraction methodology involves the detection of salient points based on image color, and these points are subsequently used for the definition of salient regions based on superpixel segmentation. From each detected salient point and region, a feature vector is calculated to describe the local color properties of the image that differentiate the abnormal from the normal tissues.

### 3.1. Salient Point Detection

**Figure 1:**
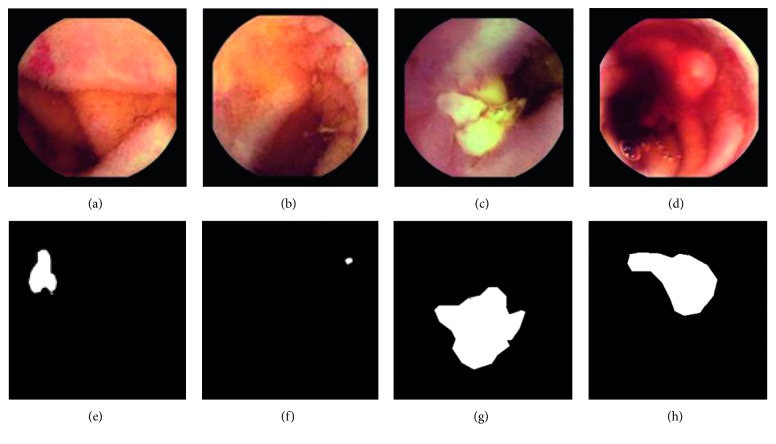
Representative images from KID dataset [20]. (a) Vascular lesion. (b) Small inflammatory lesion. (c) Large inflammatory lesion. (d) Polypoid lesion. (e–h) Detailed graphic annotations indicating the locations of the abnormalities within the respective WCE images (a–d).

A novel algorithm is proposed for the detection of salient points, specifically in WCE images. Unlike previous approaches, saliency is defined with respect to *color differences*. The proposed approach is based on the observation that within WCE images, the appearance of abnormalities may be described within a relatively small color range that is usually located on the margins of the overall color range of an image. In many cases, this range is nonoverlapping with the color range of the normal image content. By examining each WCE image separately, one may observe that the color ranges are different for each image, even for the same kind of abnormalities (Figure 1). Also, given a diverse set of WCE images, the color ranges of both the normal and abnormal content are completely overlapping. Therefore, it is not straightforward to specify a standard color range discriminating the abnormalities from normal content.

The rationale of the proposed saliency detection algorithm can be explained by the respective color histograms of WCE images. We consider that WCE images are represented in the CIE-*Lab* color space, which describes color with approximately decorrelated components [6]. The components of this space represent lightness (*L*), the quantity of red (*a *> 0) or the quantity of green (−*a *> 0), and the quantity of yellow (*b *> 0) or the quantity of blue (−*b *> 0) of a pixel. This way, color can be examined separately from lightness, which in our case varies significantly depending on the distance and the angle of the endoscope from the tissue surface. Thus, by only using the chromatic components *a* and *b*, the color information can be isolated, and an approximately illumination-invariant description of the image content may be obtained.

**Figure 2:**
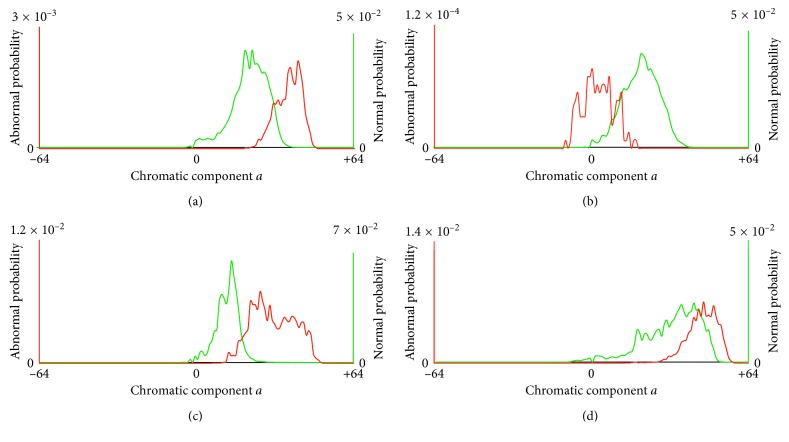
Normalized chromatic histograms of WCE images of Figure 1 (for chromatic components that have nonoverlapping regions). Histogram *H*^A^ which is estimated from abnormal image regions is represented with a solid red line, and *H*^N^ which is estimated from normal image regions is represented with a dashed green line. (a) Vascular lesion. (b) Small inflammatory lesion. (c) Large inflammatory lesion. (d) Polypoid lesion.

**Figure 3:**
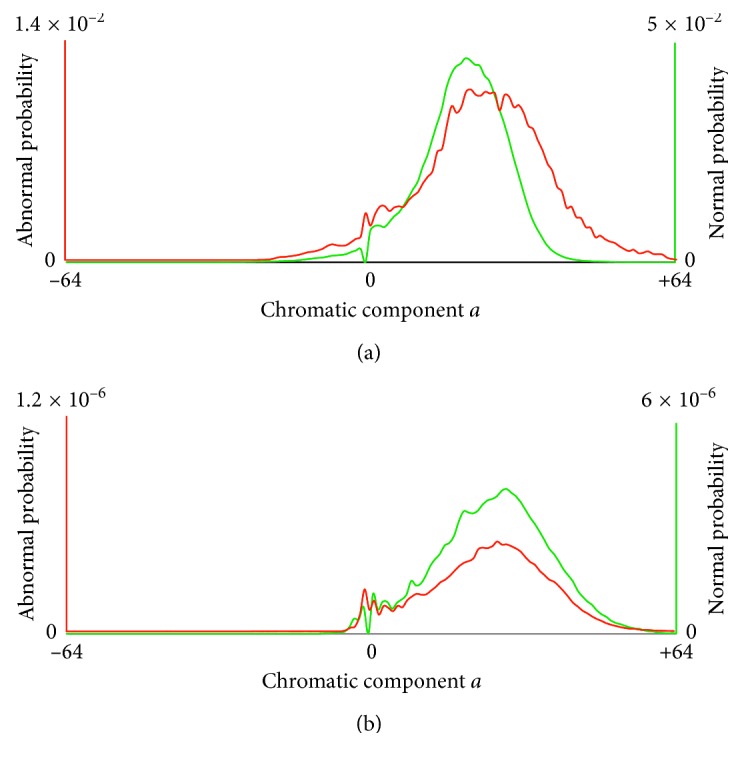
Normalized chromatic histograms estimated from all WCE images in KID dataset [20]. Histogram *H*^A^ which is estimated from abnormal image regions is represented with a solid red line, and *H*^N^ which is estimated from normal image regions is represented with a dashed green line. (a) Chromatic component *a*. (b) Chromatic component *b*.

Let *H*^A^ and *H*^N^ be the normalized histograms (probability distributions) of a WCE image for abnormal and normal regions, respectively. For the images of Figure 1, the respective histograms are illustrated in Figure 2. *H*^A^ is represented by a red line, and *H*^N^ is represented by a green line. We provide the histograms for the chromatic components, that is, *a* or *b*, of the images where a nonoverlapping range between the two histograms can be observed. For example, in Figure 2(a), a nonoverlapping region between *H*^A^ and *H*^N^ can be observed only in component *a* (in the chromatic region *a* ∈ [−9, −1]); the respective histograms of component *b* are omitted. Similarly, Figures 2(b)–2(d) present the nonoverlapping histograms of the images illustrated in Figures 1(b)–1(d). In Figure 3, the normalized histograms estimated over all images of KID dataset (Section 2) are provided, where a total overlap between the abnormal and normal chromatic ranges can be observed.

We propose a novel algorithm named SARC (Algorithm 1), which performs a Selective Aggregation of chRomatic image Components after an automatic segmentation process. This is based on the observation that abnormal image regions are usually characterized by higher positive or negative values of the *a* and *b* chromatic components. SARC produces saliency maps which emphasize on the regions that correspond to possible abnormalities.

**Figure 4:**
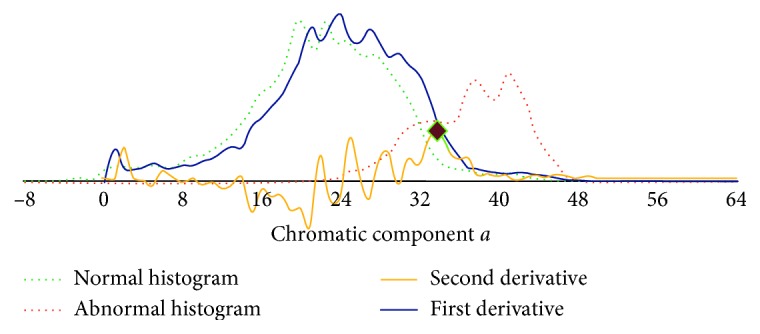
Determination of the optimal threshold *T*_*a*_^+^ (black point) based on the second derivative *R*_*a*_^+^ of the histogram estimated from chromatic component a of the WCE image of Figure 1. The application of this threshold results in Figure 5(a).

Let *I*_*Lab*_ be a *M *× *N*-pixel CIE-*Lab* input image, and *I*_a_ and *I*_b_ be the grayscale images representing *a* and *b* components of *I*_*Lab*_. This algorithm uses the histogram *H*_c_ of image *I*_c_, *c* = *a*, *b*, to determine optimal thresholds maintaining the image regions that have a higher probability to include an abnormality. It calculates the first (*r*_c_) and second derivatives (*R*_c_) of the positive (+) and negative (−) axes of *H*_c_ and determines the maximum of each of the second derivatives as an optimal image threshold for maintaining as much as discriminating information about the possible abnormal regions within the chromatic components of the image as possible. This process determines the value of the chromatic component (*a* or *b*) where the rate of the first derivative changes. Considering that the chromatic ranges of the abnormal regions are located at the margins of the histograms, this value will be the most probable one for the abnormality.  Figure 4 illustrates this concept. It can be noticed that the maximum of the second derivative corresponds approximately to the value of the chromatic component *a* with the maximum probability of the abnormality.

**Figure 5:**
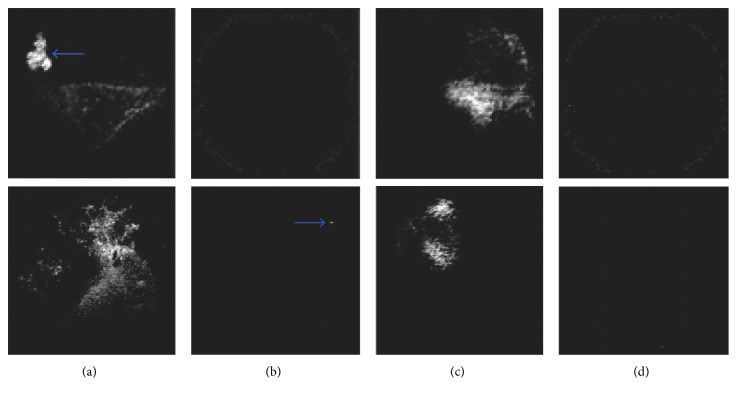
Representative output images obtained from the application of Algorithm 1 on the WCE images illustrated in Figure 1 (first row) and Figure 1 (second row) respectively. (a) *I*_a_ positive. (b) *I*_a_ negative. (c) *I*_b_ positive. (d) *I*_b_ negative. The arrows indicate the locations of the lesions. The vascular lesion of Figure 1 is clearly discriminated in the respective *I*_a_ positive image. Also the small inflammatory lesion of Figure 1 is clearly discriminated in the respective *I*_a_ negative image.

By applying the determined thresholds on the respective chromatic image components, four images are obtained. Indicative examples of such images for the cases of Figures 1 and 1 are illustrated in the first and in the second row of Figure 5, respectively. These images are subsequently filtered using a sliding window of *n *× *n* pixels, which aims to discard local nonmaxima. The final step of SARC algorithm is the aggregation of the four filtered images using the sum operator.

Algorithm 2 uses *I*_SARC_ to detect regions with significant changes in the chromatic components of CIE-Lab color space. Initially, *I*_SARC_ is sampled using concentric square windows of *s × s* and *s*/2 × *s*/2 pixels, respectively, at each pixel of *I*_SARC_ with nonzero value. These pixels are more considered as points of more interest, since they have a maximum value within their neighborhood. From these points, we define as salient ones those that are characterized by a significant change in the chromatic values of their local neighborhood. This change is calculated by the distance between the maxima and the minima extracted from the concentric square windows. This definition of saliency is inspired by the fact that such chromatic changes are usual in the neighborhoods of most abnormalities.

### 3.2. Salient Region Detection

**Figure 6:**
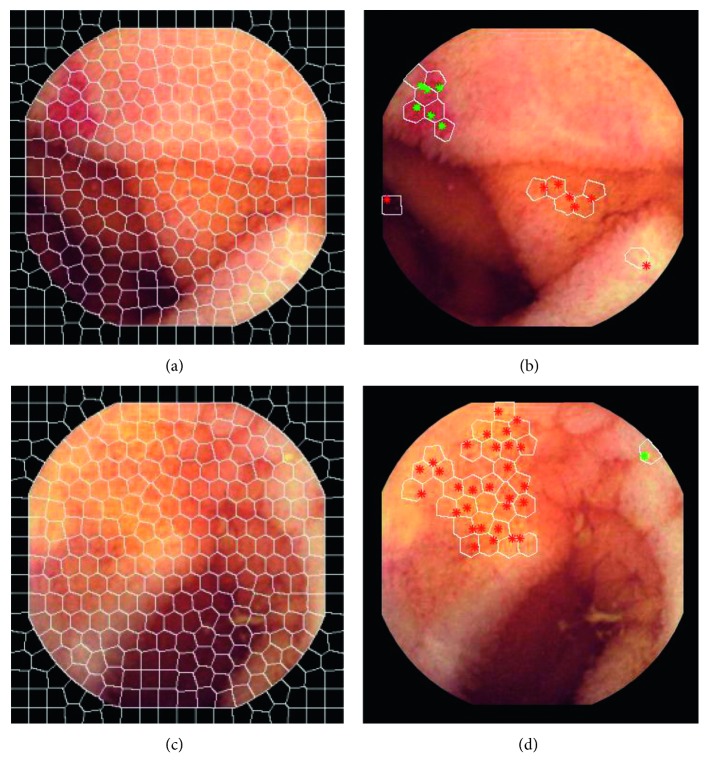
Result of SLIC algorithm on an endoscopy image (a) and superpixels with DINOSARC points (b). The images (c) and (d) show the effectiveness of saliency detection for small lesions.

The salient point detection process is followed by sampling image regions from their neighborhoods in order to estimate relevant descriptors. Instead of sampling square-shaped neighborhoods, as in [5, 6], the DINOSARC descriptors are extracted from arbitrary-shaped neighborhoods. To this end, the input images are segmented using the simple iterative linear clustering (SLIC) algorithm [21]. SLIC creates clusters of pixels defining regions of homogeneous color properties, called superpixels (Figure 6). Considering the approach we proposed in [19], the superpixels that contain at least one salient point are also characterized as salient. However, in that study, the pixel-level saliency was disregarded and the localization of abnormalities smaller than a superpixel was impossible. In this study, the pixel-level saliency defined by DINOSARC algorithm is not superseded by the region-level saliency defined by the superpixels. Each DINOSARC salient region is defined by a superpixel that includes only a single, representative salient point. If the superpixel contains a cluster of salient points, then the cluster centroid is regarded as its corresponding salient point.

### 3.3. Local and Global Color Image Descriptors

Another novel contribution of this work is that both DINOSARC salient regions and points are represented by a local color feature vector. The local feature vectors are subsequently used for the formation of feature vectors globally representing the WCE images. The feature extraction process presented in this paper is an extension of the approach we originally proposed in [6] for only local representation of square WCE image patches along with their central point.

The proposed, extended approach forms a 9-dimensional feature vector from the color components (*L*, *a*, *b*) of the CIE-*Lab* representation of a salient point, as well as the minimum and maximum values of each of the *L*, *a*, and *b* components within the DINOSARC salient region, that is, min (*L*), max (*L*); min (*a*), max (*a*); and min (*b*), max (*b*). This is inspired by the way the WCE video reviewers empirically assess the image regions for the detection of abnormalities, which, takes into account regional color differentiations [7]. By only including the minimum and maximum values from the salient regions (which are also determined by salient points derived from color differences), such differentiations can be captured.

The local image representation approach is extended by adopting the BoVW model [18] for the extraction of global features from the WCE images. This model considers that an entire image can be represented by a visual vocabulary. Such a vocabulary may be seen as a set of “exemplar” image patches (visual words), in terms of which any given image may be described. The vocabulary may be seen as a means of quantization of the feature space derived from the local feature vectors. Then, any previously unseen descriptor may be easily quantized to its nearest visual word. Thus, the DINOSARC feature vectors are used to form histograms of visual words for the representation of entire WCE images.

## 4. Results and Discussion

Experiments on the WCE images were performed to evaluate the proposed DINOSARC feature extraction methodology in comparison to the state-of-the-art using the publicly available data described in Section 2. The results obtained are organized as follows: (a) evaluation of the proposed salient point detector with respect to its capability to detect abnormalities; (b) evaluation of the proposed local descriptor for the discrimination of abnormal from normal salient regions; (c) evaluation of the proposed global descriptor for the detection of abnormal images.

### 4.1. Salient Point Detection

Prior to the application of DINOSARC algorithm, we performed a series of experiments to determine its optimal parameters. The criterion considered for this tuning process was the number of false negative images, that is, the number of images that were actually containing abnormalities, but no salient points were detected on these abnormalities. Since the salient point detection process is considered as the first step in the analysis of the WCE images, it is important to be able to detect points on abnormalities, in as many as possible (ideally in all) abnormal images.

**Figure 7:**
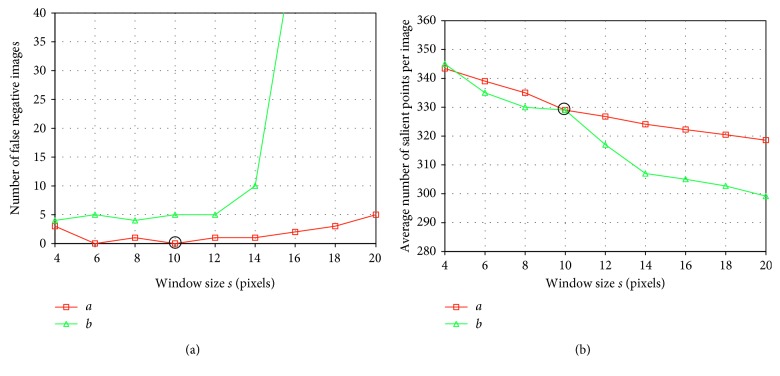
Salient point detection results for different (square) window sizes *s* × *s*. (a) Average number of salient points detected per image. (b) Number of images in which salient points have not been detected within abnormal regions (false negative).

To this end, the salient point detection performance of DINOSARC algorithm was investigated using various window sizes *s *× *s* between 4 × 4 and 20 × 20 pixels in each component of CIE-*Lab* color space. The results are illustrated in Figure 7. By using window sizes of 6 × 6 and 10 × 10 pixels in component *a*, at least one salient point was detected within the abnormalities. Among these choices, the 10 × 10 pixel window is considered preferable because it results in less salient points per image (Figure 7(b)).

**Figure 8:**
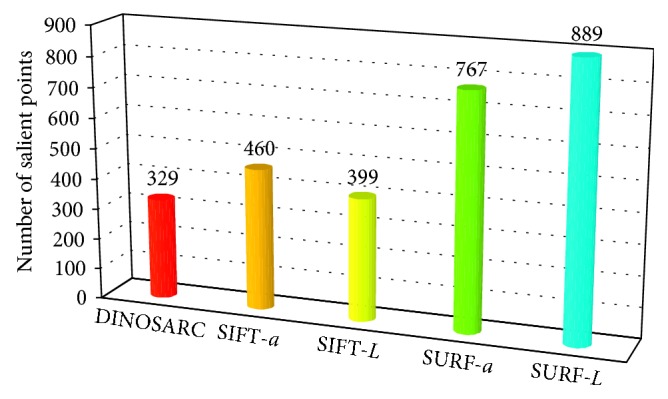
Number of salient points detected within abnormal regions over abnormal images using different methods.

The DINOSARC salient point detector was compared with the standard SIFT [22] (SIFT-*L*) and SURF [9] (SURF-*L*) algorithms, as they are typically applied on the luminance component (*L*) of images. Also, it was compared with the SURF-*a* color salient point detection method proposed in [6], where SURF was applied on component *a* of CIE-*Lab* color space. For completeness, SIFT was also tested on that color component (SIFT-*a*). The evaluation criterion for every detector was the minimum number of salient points needed in order to have the zero false negative images (Figure 8).

Further reduction of the DINOSARC salient points is achieved by the salient region detection process (Section 3.2), which results in only a single salient point per salient region. In the evaluation of DINOSARC detection algorithm, we also computed the percentage of the salient points falling on the abnormal regions of the images. The percentages of these true positive points over the total number of detected points in the image are presented in Table 1. It can be noticed that the proposed salient point detection algorithm results in more true positive points in every abnormal image than the other algorithms.

### 4.2. Salient Region Discrimination Using Local Descriptors

For the discrimination of abnormal from normal salient regions, classification experiments were performed using various local image descriptors. As a baseline to compare our DINOSARC descriptor, we considered the hue histogram of the area around each salient point. Since this descriptor is not associated with a particular salient point detection algorithm, the DINOSARC salient point detector was used. The hue histogram was quantized into 15 bins, which was the best performing one among histograms of 15 *i*, *i*=1, …, 24 bins. Also, for comparison purposes, we selected three state-of-the-art methodologies. The methodology of Yuan et al. [16], which is very recent, the methodology of Li and Meng [15], and the methodology of Iakovidis and Koulaouzidis[6], which is a predecessor of the proposed approach. The experiments were performed using the 10-fold cross validation evaluation scheme, and a Support Vector Machine (SVM) with Radial Basis Function (RBF) kernel, as a standard classifier. The classification performance was thoroughly investigated using Receiver Operating Characteristic (ROC) analysis [23]. These curves illustrate the trade-off between sensitivity and specificity for various decision thresholds. The Area Under the ROC (AUC) was estimated to be able to compare the classification performances using a single measure. The classification accuracy, the sensitivity, and the specificity are provided as additional measures facilitating comparisons.

**Figure 9:**
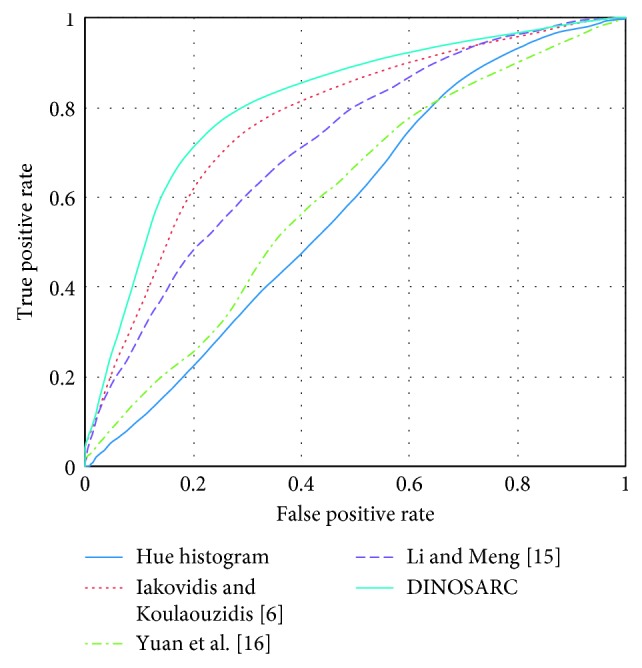
The ROCs corresponding to the AUCs reported in Table 2 for the classification of salient regions using local image descriptors.

The results are presented in Table 2. The best AUC was obtained with the proposed methodology. The respective ROCs are illustrated in Figure 9. It can be noticed that methodology of Yuan et al. provides a higher accuracy; however, this is due to the high specificity, whereas the sensitivity is very low, that is, its capability to detect positive image regions is low. Also low was the performance of the hue histogram descriptor. The second best performance was obtained by the method of Iakovidis et al.

An interpretation of these results can be based on the physical meaning of the respective descriptors. The descriptors proposed by Yuan et al. [16] and Li and Meng [15] encode the texture of an image area (both SIFT/CLBP and ULBP/wavelet transform are textural descriptors), and hue histograms encode its colors as they are perceived by humans [24]. The best performing approaches are also based on color; however, the regional minima and maxima of the opponent color components tend to provide more discriminative information about the abnormalities. The approach of Yuan et al. [16] was originally proposed for the detection of polyps, and the approach of Li and Meng [15] was proposed for the detection of tumors, including adenomas and adenocarcinomas. Texture has been a discriminative feature of polyps and tumors in several studies with flexible endoscopy images [13, 25]. However, the significantly lower resolution of WCE images limits the visibility of texture, and consequently, texture becomes less discriminative. More importantly, the database used in our experiments not only contains polyps but also several other kinds of abnormalities, for which texture may not be as discriminative as color, for example, vascular lesions.

### 4.3. Abnormal Image Detection Using Global Descriptors

**Figure 10:**
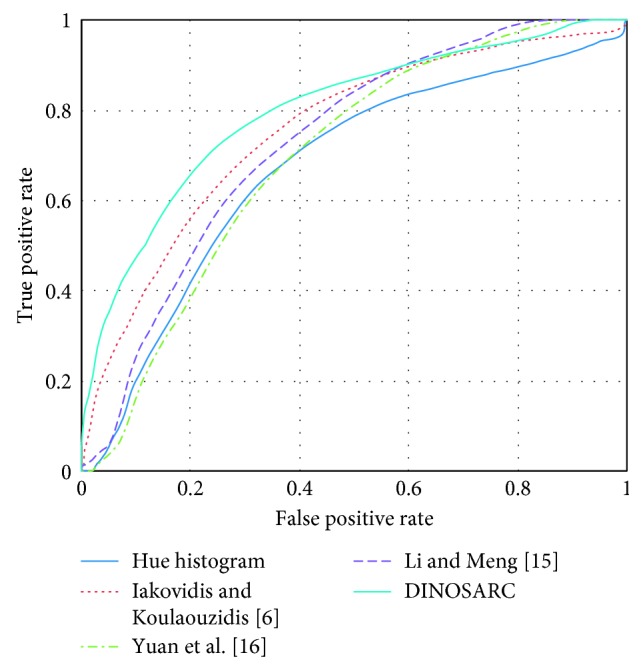
The ROCs corresponding to the AUCs reported in Table 3 for the classification of WCE images using global image descriptors.

Experiments were performed for the investigation of the classification performance of entire WCE images using the DINOSARC features. For image representation, global features were extracted using the BoVW model. The BoVW model was constructed with a range of visual vocabulary sizes in the range from 500 to 700 words. The experiments were performed using the 10-fold cross validation evaluation scheme and an RBF-SVM classifier.  Table 3 summarizes the results obtained. The proposed DINOSARC features achieved better results from the other methods. The respective ROCs are illustrated in Figure 10. As in Section 4.2, considering the AUCs, the method of Iakovidis and Koulaouzidis was ranked second, the method of Li and Meng [15] was ranked third, the method of Yuan et al. was ranked fourth, and the lowest classification performance was obtained by the hue histograms.

## 5. Conclusions

We presented DINOSARC, a color feature extraction methodology for WCE image analysis. The proposed methodology aims to the discrimination of various abnormal tissues from normal image contents. Major contributions of this study include the following:

- A novel salient point detection method, which considers saliency with respect to color differences observed in abnormality regions.
- A novel definition of regional saliency based on superpixel segmentation that extends the approach we previously proposed for bleeding detection [19]. The extension relies on the fact that region-level saliency is defined based on DINOSARC salient points and that point-level saliency is preserved to enable the localization of smaller abnormalities.
- A novel descriptor, which extends the descriptor we proposed in [6] by applying the calculations on an arbitrarily shaped local region defined by a salient superpixel.
- The proposed methodology was applied for both supervised and weakly-supervised detection of abnormalities in a rich publicly available dataset. The supervised approach was based on the proposed local descriptors, and the weakly-supervised approach was based on global image descriptors derived from the local ones by application of the BoWV model.

The results showed that the proposed methodology can be more efficient and more effective than relevant state-of-the-art methods for the detection of abnormal images. More, specifically:

- The proposed salient point detection approach results in a smaller number of salient points, which are more likely to fall within regions of abnormality than other current approaches
- The proposed local image descriptors result in better discrimination of the abnormalities from the normal image contents
- The global image descriptors enable more accurate detection of the abnormal images in the WCE dataset

Future research directions include investigation of methods towards further decreasing the total number of salient points, further improvement of the discrimination capability of the image descriptors, and the extension of the experimentation to entire video sequences.

## Figures and Tables

**Algorithm 1:**
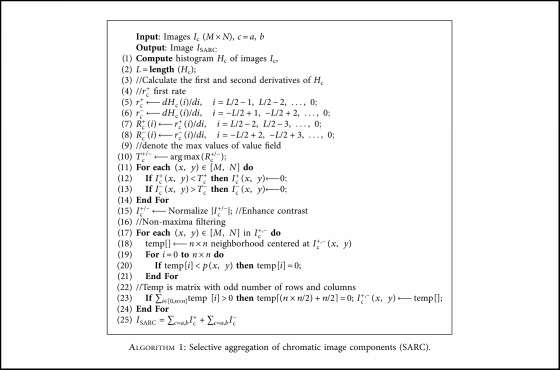
Selective aggregation of chromatic image components (SARC).

**Algorithm 2:**
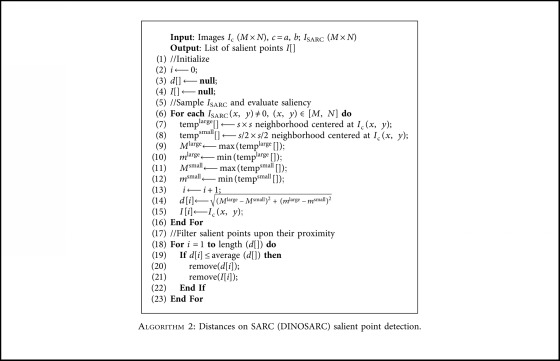
Distances on SARC (DINOSARC) salient point detection.

**Table 1:** True positive points for each image.

Algorithm	Salient points (%)
DINOSARC	20
SIFT-*L*	14
SURF-*L*	11
SIFT-*a*	15
SURF-*a*	12

**Table 2:** Classification results of salient regions using local image descriptors.

	Hue histogram	Iakovidis and Koulaouzidis [6]	Yuan et al. [16]	Li and Meng [15]	DINOSARC
AUC	0.584	0.774	0.606	0.718	**0.813**
Accuracy	0.671	0.772	**0.874**	0.698	0.809
Sensitivity	0.833	0.699	0.142	0.432	0.680
Specificity	0.232	0.782	**0.974**	0.829	0.814

**Table 3:** Classification results of WCE images using global image descriptors.

	Hue histogram	Iakovidis and Koulaouzidis [6]	Yuan et al. [16]	Li and Meng [15]	DINOSARC
AUC	0.684	0.774	0.701	0.754	**0.815**
Accuracy	0.730	0.786	0.746	0.751	**0.818**
Sensitivity	0.391	0.496	0.406	0.358	**0.512**
Specificity	0.871	0.890	0.884	0.870	**0.908**

## Data Availability

The database of WCE images described in Section 2 is publicly available at http://is-innovation.eu/kid/, Image Collection “Dataset 2.”
